# Analysis of temporal diversification of African Cyprinidae (Teleostei, Cypriniformes)

**DOI:** 10.3897/zookeys.806.25844

**Published:** 2018-12-13

**Authors:** Mariam I. Adeoba, Kowiyou Yessoufou

**Affiliations:** 1 Department of Zoology, University of Johannesburg, Kingsway Campus PO Box 524, Auckland Park 2006, South Africa University of Johannesburg Johannesburg South Africa; 2 Department of Geography, Environmental management and Energy studies, University of Johannesburg, Kingsway Campus PO Box 524, Auckland Park 2006, South Africa University of Johannesburg Johannesburg South Africa

**Keywords:** climate change, extinction, fish, geological rift, speciation

## Abstract

Recent evidence that freshwater fishes diversify faster than marine fishes signifies that the evolutionary history of biodiversity in freshwater system is of particular interest. Here, the evolutionary diversification events of African Cyprinidae, a freshwater fish family with wide geographic distribution, were reconstructed and analysed. The overall diversification rate of African Cyprinidae is 0.08 species per million year (when extinction rate is very high, i.e., ε = 0.9) and 0.11 species per million year (when ε = 0). This overall rate is lower than the rate reported for African Cichlids, suggesting that African freshwaters might be less conducive for a rapid diversification of Cyprinidae. However, the observed diversification events of African Cyprinidae occurred in the last 10 million years. The temporal pattern of these events follows a non-constant episodic birth-death model (Bayes Factor > 28) and the rate-constant model never outperformed any of the non-constant models tested. The fact that most diversification events occurred in the last 10 million years supports the pattern reported for Cyprinidae in other continent, e.g., Asia, perhaps pointing to concomitant diversification globally. However, the diversification events coincided with major geologic and paleo-climatic events in Africa, suggesting that geological and climatic events may have mediated the diversification patterns of Cyprinidae on the continent.

## Introduction

The standing biodiversity, i.e., the diversity of life that we are witnessing today, is the result of countless speciation and extinction events that have occurred in the past (Jablonski 1995; May et al. 1995; Niklas 1997). An important body of literature has been devoted to explain not only what drives these macro-evolutionary events (ecological or stochastic forces) but also their temporal dynamics (Morrison et al. 2004; Williams and Reid 2004; Xiang et al. 2005; Kozak et al. 2006; Weir 2007; Phillimore and Price 2008). These studies explored specific questions: i) did macro-evolutionary events occur at a constant rate with species accumulating exponentially over time? Alternatively, ii) did they occur at non-constant rates with bursts of speciation at the origin (owing to availability of empty niches) followed by a rate decrease over time (adaptive radiation)?

In macro-evolutionary studies, fossil records are believed to track better the temporal dynamic of species accumulation (Raup 1976; Jablonski 1995; Niklas 1997). However, a number of limitations preclude a common or frequent use of fossil records. First, we do not always have fossil records for several taxonomic groups of interest (Raup 1976; Jablonski 1995; Niklas 1997; Peters and Foote 2002). This is especially the case for taxonomic groups with soft body parts or those that occur in arid environments because they are rarely fossilized. Second, it is difficult to identify punctuated events (e.g., mass extinction) in fossil records, and as a result, the analysis of fossil records “can give the impression that the diversity of life has increased inexorably through time” (Crisp and Cook 2009).

In the face of the limitations of fossil records for macro-evolutionary studies, DNA-based phylogenies provide a commonly used alternative approach (e.g., Harvey et al. 1994; Rüber and Zardoya 2005). Most recent studies employed the phylogenetic approach to investigate the dynamics of species accumulation over time, and these dynamics are best interpreted when represented graphically in the form of lineages-through-time plot (LTT plot). Various theoretical scenarios of LTT plots are plausible as described in Crisp and Cook (2009). First, the tempo and mode of species accumulation can be constant over time corresponding to a linear semi-log LTT plot due to a constant ratio birth/death through time. Second, the pattern can depart from a linear semi-log LTT plot showing a concave or convex line as a result of single rate decrease or increase, respectively. Third, a pattern of early rapid radiation that later slows down can also be observed. This is known as adaptive radiation and is thought to be driven by ecological opportunities (i.e., the need to fill empty niches trigger a rapid radiation, which slows down over time as the proportion of empty niches decrease with time). Characteristics of LTT plot corresponding to adaptive radiation are steep slope at the origin and the slope flattens progressively. Finally, when the LTT plot has an anti-sigmoidal shape, this is indicative of a constant radiation punctuated by mass extinction events (Crisp and Cook 2009).

In the literature, the adaptive radiation is the most commonly reported scenario irrespective of the taxonomic groups studied (Shaw et al. 2003; Kadereit et al. 2004; Machordom and Macpherson 2004; Morrison et al. 2004; Williams and Reid 2004; Xiang et al. 2005; Kozak et al. 2006; Weir 2006; Phillimore and Price 2008; Seehausen 2015). Nonetheless, it is important to highlight that these studies are biased not only towards plants but specifically towards plant groups of exceptionally high diversification rate (Baldwin and Sanderson 1998; Richardson et al. 2001; Verboom et al. 2003; Klak et al. 2004; Kay et al. 2005; Hughes and Eastwood 2006; García-Maroto et al. 2009; Valente et al. 2010). In the animal kingdom, similar high radiations were also reported in the Cambrian era (see Rokas et al. 2005) but Rokas et al.’s study was very broad as it focused on Metazoa in general. Even studies that explored more specifically the diversification patterns of vertebrate also focused on groups that showed relatively high diversification rate (e.g., birds, Moyle et al. 2009). For fish, particular attention has been given to the African Cichlids (McCune 1997; Verheyen et al. 2003; Day et al. 2008), again because this group of fish showed spectacular diversification rates (8.29–62.15 species per million years in the Lake Victoria; Verheyen et al. 2003).

However, the phylogenetic approach too has some limitations, with the most commonly cited limitation being the lack of complete DNA data for most lineages of interest. For example, in the vertebrate group, we only have DNA sequences (COI) for 67% of extant bird species (Jetz et al. 2012), 55% of mammals (Bininda-Emonds et al. 2007), 45% of squamate reptiles (Pyron et al. 2013) and the lowest proportion of available DNA sequences is for fishes (27%; Rabosky et al. 2013). Consequently, we have a poor understanding of the evolutionary history and diversification patterns of several vertebrate groups due to unavailability of complete DNA data. Although several statistical approaches have been proposed to simulate complete sampling (e.g., see Pybus and Harvey 2000), a complete DNA- and/or taxonomic-based phylogeny would always be better for ecological and evolutionary studies than simulated phylogenies (see Rabosky 2015). Interestingly, owing to the ongoing global DNA barcoding campaign, an impressive volume of DNA sequences is increasingly made available in public repositories (www.boldsystems.org) and these sequences can be used for taxonomic, ecological, and evolutionary studies. Even when complete DNA sequences are not yet available for a particular taxonomic group of interest (e.g., African Cyprinidae), a recent improvement in methodological approaches now allows the reconstruction of a comprehensive phylogenetic tree (see Thomas et al. 2013) without any simulation. Thomas et al.’s approach (see details in Material and Method Section) combines taxonomic information with the available incomplete DNA sequences to assemble a complete phylogenetic tree. The resulting phylogeny from Thomas et al.’s approach is showed multiple times to be suitable for the analysis of diversification rates and evolutionary processes (e.g., Jetz et al. 2012) especially when trait data are not involved (see Rabosky 2015).

In the present paper, Thomas et al.’s approach was used to assemble a complete phylogeny for the African Cyprinidae. A higher proportion of the African freshwater ichthyofauna belongs to the family Cyprinidae after the cichlids (Skelton et al. 1991). For example, 24 genera and 539 species of Cyprinidae are recognized in Africa (Froese and Pauly 2017). They are distributed from the northern to southern Africa, with their mature sizes ranging from small (30 mm SL) to larger size (900 mm SL) (Skelton et al. 1991). The aim of the present Chapter was to understand the evolutionary processes that shaped the current diversity of the fish family Cyprinidae in Africa. Specifically, four questions were investigated. First, what is the overall rate of the evolutionary processes (speciation and extinction events) that led to the observed diversity of Cyprinidae in Africa? ii) How does this rate compare to the rates reported for African Cichlids? iii) Have diversification rates of African Cyprinidae changed significantly through time? iv) Is there evidence that African Cyprinidae experienced mass extinction events?

## Materials and methods

### Assembling a fully sampled phylogeny of the African Cyprinidae

The recent approach of Thomas et al. (2013) was used to assemble a complete phylogeny, as we do not have a complete matrix of DNA sequences for all species. This approach requires taxonomic information and DNA data. The DNA data used are the COI matrix that we recently assembled and published (see details in Adeoba et al. 2018). These DNA sequences were used to first reconstruct a constraint tree. With regard to DNA data, three types of species (types 1, 2 and 3) are distinguished: “type 1 species” are species for which COI sequences are available; “type 2 species” are species for which COI sequences are missing but they are congeners of type 1; “type 3 species” have no COI sequences and are not congeners of type 1. In this study, there are 138 type 1 species, 388 type 2 species, and 13 type 3 species.

To assemble the constraint tree, an XML file was generated using the COI sequences of the type 1 species, in the program BEAUTi, and this file was used to reconstruct a dated constraint tree based on a Bayesian MCMC approach implemented in the BEAST program. Next, the GTR + I + Γ model was selected as the best model of sequence evolution based on the Akaike information criterion evaluated using MODELTEST (Nylander 2004). In addition, a Yule process was selected as the tree prior with an uncorrelated relaxed lognormal model for rate variation among branches. Also, the COI sequences of the following species were used as outgroups and for calibration purpose (He et al. 2008; Tang et al. 2010; Wang et al. 2012): *Barbonymusaltus* (Günther, 1868), *Barbonymusschwanenfieldii* (Bleeker, 1854), *Barbusbarbus* (Linnaeus, 1758), *Carrassiusauratus* (Linnaeus, 1758), *Carrassiusgibelio* (Bloch, 1782), *Gyrinocheilusaymonieri* (Tirant, 1883), *Hybognathusargyritis* Girard, 1856, *Myxocyprinusasiaticus* (Bleeker, 1864), *Paramisgurnusdabryanus* Dabry de Thiersant, 1872, *Phoxinusphoxinus* (Linnaeus, 1758), *Pseudorasboraparva* (Schlegel, 1842), *Rhinichthysumatilla* (Gilbert & Evermann, 1894), *Tincatinca* (Linnaeus, 1758) and *Vimbavimba* (Linnaeus, 1758). For calibration purpose, following Wang et al. (2012) and Cavender (1991), the root node of Cyprinidae was constrained to 55.8 million years (My) and the split between *Tinca* and the modern leuciscins was constrained to 18.0 My. In addition, the lineage *Barbus* was calibrated to 13 My following Zardoya and Doadrio (1999). Monte Carlo Markov chains were run for 50 million generations with trees sampled every 1000 generations. Log files, including prior and likelihood values, as well as the effective sample size (ESS) were examined using TRACER (Rambaut and Drummond 2007). ESS values were all > 200 for the age estimates. The first 25% of the resulting 50,000 trees were discarded as burn-in, and the remaining trees were combined using TREEANNOTATOR (Rambaut and Drummond 2007) to generate a maximum clade credibility (MCC) tree (the constraint tree).

To integrate the types 2 and 3 species into the constraint tree, a simple taxon definition file that lists all three types of species along with their taxonomic information (here genus names) was formed. Using the constraint tree and the taxon definition file, an MrBayes input file was first generated as implemented in the R library PASTIS (Thomas et al. 2013), and then a dated complete phylogeny using MrBayes 3.2. (Ronquist and Huelsenbeck 2003) was reconstructed under a relaxed-clock model with node-age calibrations indicated above.

In the 10,000 resulting trees, the topology of species with DNA-sequences remains fixed, and the unsampled species (types 2 and 3) were assigned randomly within their genera. In an early study, Kuhn et al. (2011) demonstrated that similar approach to that of Thomas et al. (2013) generates conservative placements of types 2 and 3 species with respect to divergence times and diversification rate estimation, which is, following Rabosky (2015), a significant advance for diversification studies.

### Data analysis

All analyses were done in R (R Development Core Team 2011). To understand how the observed diversity of African Cyprinidae was accumulated over time, multiple approaches described below were used.

First, the net diversification rate (speciation minus extinction) was estimated using Magallón and Sanderson’s method (Magallón and Sanderson 2001) implemented in the R library GEIGER (Harmon et al. 2007) under two scenarios: no extinction (ε = 0) and high extinction rate (ε = 0.9).

Second, the observed net diversification rates were compared to those reported for Cichlids in various African lakes (Lake Malawi and Lake Victoria).

Third, to assess whether the diversification rates of African Cyprinidae have changed significantly through time, the gamma statistic (Pybus and Harvey 2000), the LTT (Lineage-Through-Time) plot and several evolutionary models were tested on the African Cyprinidae dataset. The value of gamma was calculated on the phylogenetic tree of Cyprinidae using the R package LASER (Rabosky 2006). If gamma < 0, this implies a decreasing speciation of Cyprinidae over time whereas gamma > 0 is indicative of an increasing speciation toward the present day (Pybus and Harvey 2000).

To test if the value of gamma departs significantly from zero, the observed value of gamma was compared, using confidence interval, to the expected value of gamma under a constant-rate birth-death model. To this end, an MCMC (Markov chain Monte Carlo) simulation was performed to estimate the posterior probability distribution of gamma under this constant-rate model. Specifically, the constant-rate birth-death model was parameterized by drawing rate parameters from the joint posterior densities inferred from the phylogenetic tree of Cyprinidae. This parameterized model was used to simulate 1000 phylogenies, and these simulated phylogenies were used to calculate the expected value of gamma. Then, the observed value of gamma was compared to the posterior-predictive distribution of the expected value of gamma. If the observed value falls near the centre of the simulated distribution, then the diversification rates of African Cyprinidae are constant over time. If not, it means that the diversification of the African Cyprinidae has significantly changed over time (Höhna et al. 2015), i.e., some diversification shifts have occurred.

In addition, the 1000 phylogenies that were simulated were used to reconstruct the posterior-predictive distribution of the corresponding LTT plots (1000 simulated LTT plots). The observed LTT plot for African Cyprinidae was then reconstructed and compared to the simulated LTT plots. If the observed LTT plot falls within the simulated LTT plots, this means that the diversification rate of African Cyprinidae has been constant over time. Otherwise, the diversification of African Cyprinidae has experienced some evolutionary shifts. Next, the observed LTT plot was also compared to the various scenarios predicted and summarized above in the Introduction (Crisp and Cook 2009). Furthermore, the evolutionary models that explain the diversification patterns depicted by the observed LLT plot of the African Cyprinidae were identified. The models tested include a constant-rate birth-death model and three rate-variation models. The rate-variation models include a birth-death model with an exponentially decreasing speciation rate, a birth-death model with piecewise-constant rates (i.e., rates of speciation and extinction change over time but the diversification rate remains constant; Höhna 2015) and a birth-death model of evolution punctuated by a mass-extinction event. The best of these models was selected based on Bayes Factors (Baele et al. 2013) whose values were interpreted following Jeffreys (1961) (Suppl. material 1: Table S1).

Finally, to investigate whether African Cyprinidae experienced some mass extinctions events (if so, when), the CoMET [Compound Poisson Process (CPP) on Mass Extinction Time) approach was used (May et al. 2016). This approach has the advantage of being able not only to fit all possible birth-death models to the data at hand but also to specifically model mass extinction events. Briefly explained, the CoMET approach treats the number of speciation-rate shifts, extinction-rate shifts, mass-extinction events, as well as the parameters associated with these events as random variables, and then estimates their joint posterior distribution. For this analysis, hyperpriors was set both *a priori* and empirically as implemented in the R package TESS (Höhna et al. 2015). The results of *a priori* hyperpriors are reported below as they are similar to that of the empirically set priors.

## Results

The phylogenetic tree of African Cyprinidae is presented in a study that is currently under review and provided here as Suppl. material 1. Based on this tree, the overall diversification rate of African Cyprinidae is 0.08 species per million year (when extinction rate is very high, i.e., ε = 0.9) and 0.11 species per million year (when ε = 0). This overall rate is lower than the rate reported for African Cichlids (see details in Discussion below).

**Figure 1.**
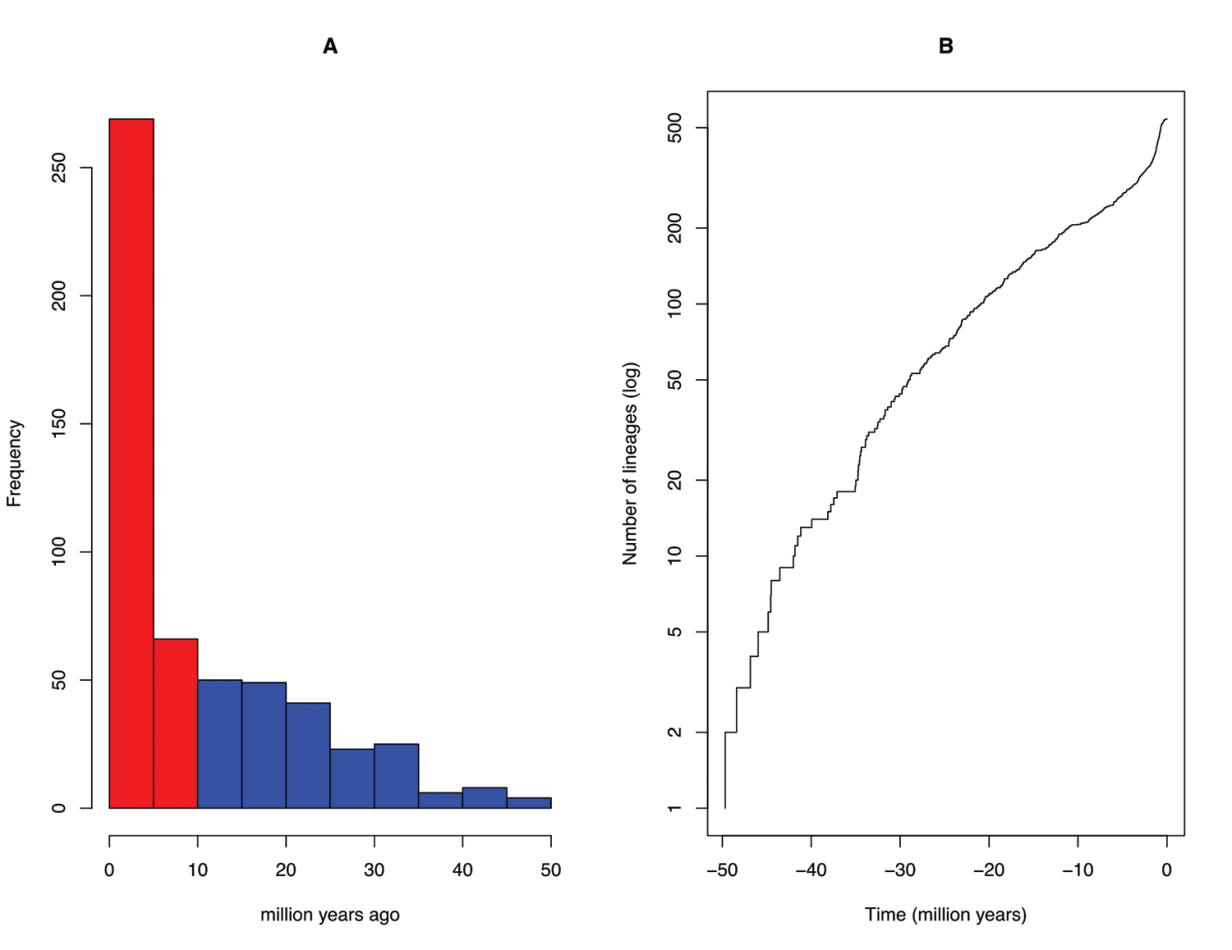
Diversification patterns of African Cyprinidae. **A** Histogram depicting the frequency of branching time on the phylogeny of African Cyprinidae; red colour shows the most frequent branching events which occurred the last 10 million years; blue colour indicates earlier branching events i.e., prior to 10 million years ago **B** lineage-through-time plot of the phylogeny of African Cyprinidae.

Most diversification events occurred in the last 10 million years (my) (Figure 1A), and the temporal pattern of these events, represented as an LTT plot (Figure 1B), follows an anti-sigmoidal shape, which is indicative of an overall constant diversification punctuated by a mass extinction event.

**Figure 2.**
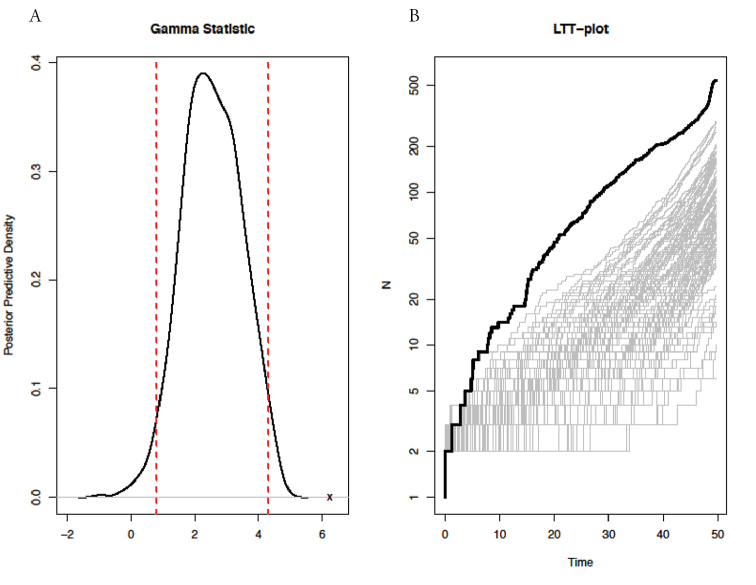
Comparative analysis of the fit of observed diversification pattern of African Cyprinidae to the constant-rate birth-death model using posterior-predictive simulation. Left panel (**A**): The posterior-predictive distribution for the gamma statistic; the dashed red lines indicate the 95% credible interval, and the “x” indicates the location of the value of the observed gamma statistic. Right panel (**B**): Lineage-through-time plot for the simulated phylogenies (grey) and for the phylogeny African Cyprinidae (bold black).

To assess whether there was a significant rate variation over the diversification period, the gamma statistic was first calculated (gamma = 6.23), and this positive gamma value (which is indicative of an increase diversification rate over time) is significantly different from the expected gamma under a rate-constant diversification model (Confidence Interval CI = 0.80–4.31; Figure 2A). This suggests that a non-constant diversification model is best suitable to explain the accumulation of African Cyprinidae over time. This result is also supported by the comparative analysis of the observed LTT plot of the African Cyprinidae with the simulated LTT plots under a rate-constant diversification (Figure 2B). This comparative analysis clearly showed that the observed LTT plot is different from the expected ones (Figure 2B).

Given this evidence of non-constant diversification, the next step was to identify the best model for the diversification pattern of African Cyprinidae. The non-constant episodic birth-death model was decisively supported (see BF interpretation in Table 1) as the best model explaining the diversification pattern of the African Cyprinidae (BF > 28) and the rate-constant model never outperformed any of the non-constant models tested (Table 2).

**Figure 3.**
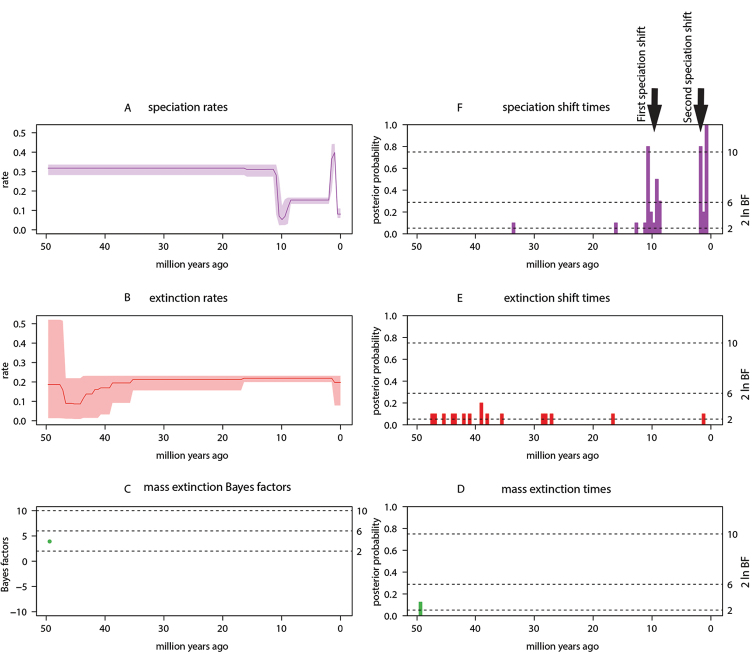
Summary of all evolutionary events (**A–F**) reported in this study. This summary was presented using the Compound Poisson Process (CPP) on Mass Extinction Time (CoMET) model. Diversification hyperpriors are specified *a priori* and empirically. Result reported are for priors set a priori as this does not differ from when empirical priors were set.

Finally, the overall evolutionary events that shaped the diversification of African Cyprinidae are summarized in Figure 3. This figure showed that the speciation rate (Figure 3A) was roughly constant to 0.3 species per million year over the first 40 my. However, within the last 10 my, the following rate variations were observed. A first and sudden rate decrease to ~0.05 species/my occurred around 10 million years ago (Ma), then this rate increased almost simultaneously to 0.15 where it remains constant until ~ 2 Ma before a sudden rate increase to 0.4 occurred followed by a punctual decrease to 0.1 around 1–0 Ma (Figure 3A). Nonetheless, 50 Ma, the extinction rate that was at 0.2, decreased to 0.1 around 45 Ma but increased steadily to 0.2 until ~ 37 Ma and remained constant to the present (Figure 3B). There was only one but not decisive mass extinction (Figures 3C, D) and 12 non-significant extinction shifts (Figure 3E). However, there was five speciation shifts, of which two are significant: the first at ~10 Ma (rate decrease) and ~2 Ma (rate increase; Figure 3F).

**Table 1.** Interpretation of Bayes factors following Jeffreys (1961). Abbreviations: M_0_ Model 0; M_1_ model 1, BF Bayes Factor.

Interpretations	BF(M_0_,M_1_)	ln(BF(M_0_,M_1_))	log10(BF(M_0_,M_1_))
Negative value is a support for model M_1_	<1	<0	<0
Barely M_0_ worth mentioning	1 to 3.2	0 to 1.16	0 to 0.5
Substantial support for model M_0_	3.2 to 10	1.16 to 2.3	0.5 to 1
Strong support for model M_0_	10 to 100	2.3 to 4.6	1 to 2
Decisive support for model M_0_	>100	>4.6	>2

**Table 2.** Bayes Factor (BF) values calculated for each pair of birth-death models tested on the phylogeny of African Cyprinidae. Abbreviations: ConstBD = constant-rate birth-death model; DecrBD = continuously variable-rate birth-death model; EpisodicBD = episodically variable-rate birth-death model, and; MassExtinctionBD = explicit mass-extinction birth-death model. The interpretations of these values should be done in comparison with the reference values in Table 1.

Model_0_	Model_1_	BF
EpisodicBD	ConstBD	28.635868
DecrBD	ConstBD	20.886607
EpisodicBD	MassExtinctionBD	20.276659
DecrBD	MassExtinctionBD	12.527398
MassExtinctionBD	ConstBD	8.359210
EpisodicBD	DecrBD	7.749261
ConstBD	ConstBD	0.000000
DecrBD	DecrBD	0.000000
EpisodicBD	EpisodicBD	0.000000
MassExtinctionBD	MassExtinctionBD	0.000000
DecrBD	EpisodicBD	-7.749261
ConstBD	MassExtinctionBD	-8.359210
MassExtinctionBD	DecrBD	-12.527398
MassExtinctionBD	EpisodicBD	-20.276659
ConstBD	DecrBD	-20.886607
ConstBD	EpisodicBD	-28.635868

## Discussion

The subfamily Labeoninae is embedded within the subfamily Cyprininae on the phylogenetic tree presented in Suppl. material 1 (see paper in review). The topology our tree puts in question early treatments of Labeoninae (Thai et al. 2007; Tang et al. 2009; Zheng et al. 2010) but supports the most recent treatment of Yang et al. (2015) who reported that the former Labeoninae is actually Cyprininae.

Using this phylogenetic tree, we found that the diversification rate of African Cyprinidae, even in the absence of extinction, is 0.11 species/ Myr, which is far lower than the rates reported for the Cichlids in various Lakes on the same continent. For example, in the Lake Malawi, McCune (1997) reported a rate between 3.00–5.99 species/Myr. In the Lake Victoria, an unusually high rate of 8.29–62.15 species/Myr was reported (Verheyen et al. 2003) whereas a latter study found that African Cichlids radiated in the Lake Tanganyika with a rate six time slower (1.67–2.71) than the rates in Lakes Malawi and Victoria (Day et al. 2008). The particularly lower radiation rate found in the present study suggests that the environment of African freshwaters is certainly less conducive for the radiation of Cyprinidae. For example, the rapid radiation of Cichlids and perhaps many other fish groups in African Lakes may have caused these groups to be the first to occupy much of the available ecological niches (Fryer and Iles 1972; Kornfield and Smith 2000), thus leading to strong competitions between these groups and Cyprinidae. Such competitive interactions may drive the low radiation of Cyprinidae as predicted in the density-dependent radiation model (McPeek 2008). However, even the rapid radiation of African Cichlids in the last 150,000 years (potentially promoted by hybridization; Meier et al. 2017; see also review in Seehausen 2015), could perhaps be an exception to the global Cichlids radiation, as a rate-constant radiation was instead reported for the South-American Cichlids (Hulsey et al. 2010). What is the temporal diversification pattern for African Cyprinidae?

All analyses performed in this study pointed to a non-constant diversification for African Cyprinidae. Specifically, the episodically variable-rate birth-death model was the best model found for the diversification pattern of African Cyprinidae. This model suggests that some decisive rate shifts have occurred during the speciation and extinction events of African Cyprinidae but, between two consecutive rate shifts, the diversification rate was constant (Stadler 2011; Höhna 2015). Indeed, as showed in Figures 3A, F, two decisive rate shifts were observed in the last 10 million years (see also Bloom et al. 2013 for New World freshwater fishes), and these were interspersed by constant radiations. The first constant speciation was before the last 10 million years and the second constant radiation occurred between 10 and 2 million years ago. In addition, there was evidence for 12 extinction events (Figure 3E), confirming frequent extinction events reported in freshwaters; e.g., extinction rates in freshwaters are 60 times greater than in marine waters (Bloom et al. 2013). However, none of the 12 extinction events found for African Cyprinidae was statistically decisive (see Table 1 for interpretation of BF).

The observed rate shifts can only be understood if analysed within the African context of geological and paleo-climatic events (Danley et al. 2012). Geological events are well established as drivers of vicariant speciation especially in freshwaters (Burridge et al. 2006; Albert and Carvalho 2011). For example, vicariance-promoting diversifications were reported in freshwater fishes (including Cyprinidae) within the south-western Cape Floristic Region during the Miocene-Pliocene transition (Chakona et al. 2013).

**Figure 4.**
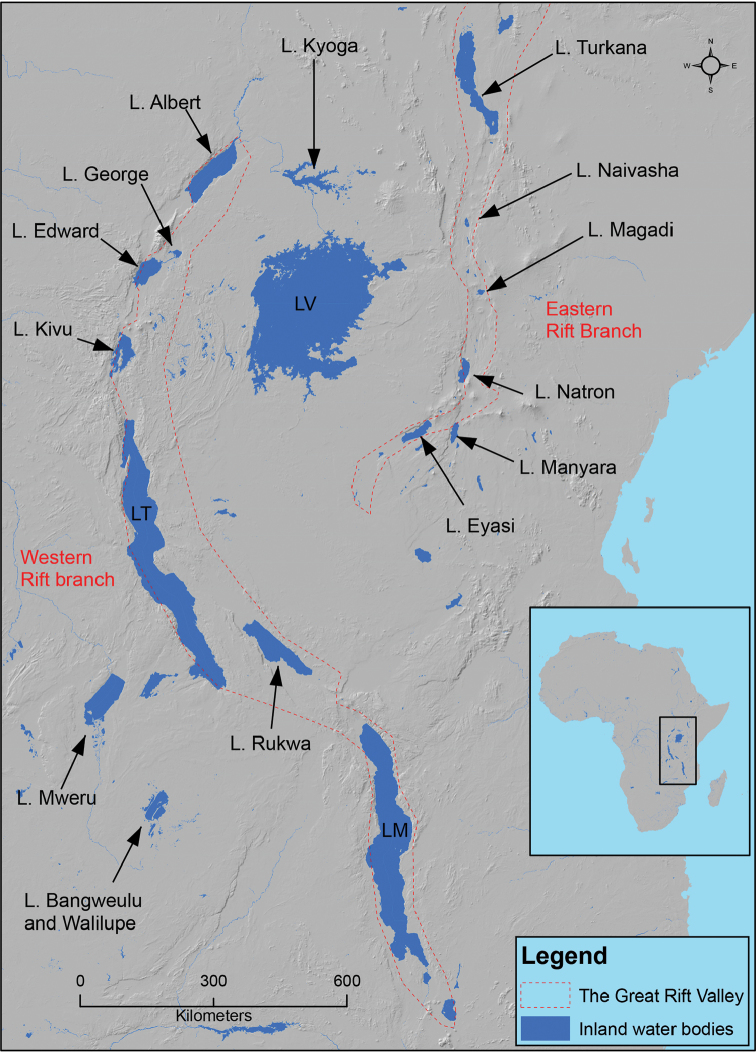
The Great Rift Valley used as a model river system to interpret the diversification of African Cyprinidae. Abbreviations: LV, Lake Victoria; LT, Lake Tanganyika, LM, Lake Malawi.

We used the example of the Great Lakes (Figure 4) as a model system to interpret our results, as this is a major river system that has been very well studied on the continent. These geological events first drive the formation of some major freshwater ecosystems in Africa, e.g., the Great Lakes (Figure 4), known as key “cradles” and “museums” of fish diversity (Danley et al. 2012). These events include shift of tectonic plates and opening of rifts, e.g., the East Africa rift system (Figure 4). This system, in particular, is made of eastern and western rifts (Nyblade and Brazier 2002). The eastern rifting started 30–35 Ma (Roberts et al. 2012) and generated some stress that was later transported westward, resulting in the creation of the western branch of the rift ~ 12–10 Ma (Roberts et al. 2012), a period that coincided with the first sudden decrease in speciation rate of African Cyprinidae (Figure 3A, F). This suggests that the geological rifting and the inherent stress likely mediated the speciation rate decrease. Later, the Lakes Tanganyika and Malawi were created (Ebinger et al. 1989; Mortimer et al. 2007); both experienced rifting around 9–12 Ma and 8–12 Ma (Cohen et al. 1993), respectively, and this may have further contributed to the observed decrease of fish speciation. During the same period, the Lakes underwent an extension northward and southward (Talbot and Williams 2009), causing the opening of new ecological niches that may have triggered the rapid speciation observed immediately after the rate decrease (between 10 and 2 Ma; Figure 3F).

In addition, there has been an extension and uplift concurrently with the rifting causing a back-ponding between the eastern and the western rifts, creating the Lake Victoria (Johnson et al. 1996; Nicholson 1996) (Figure 4). This new Lake, the largest freshwater lake in the tropics and the second largest in the world, provided new ecological niches. The fact that this lake was formed around 1.6–0.40 Ma (Danley et al. 2012), a period that coincided with the significant speciation-rate increase of African Cyprinidae, may have triggered the speciation shift.

What’s more, the dynamic of the African paleoclimate over the last 10 million years (Kingston et al. 2002) may have also mediated the diversification patterns. Around 8–10 Ma, a period that corresponds to the first significant speciation shift, African climate was humid (Cerling et al. 2011), and then became arid around 7–5 Ma (Cerling et al. 1997). Around 5–3 Ma, the climate became warmer and wetter across Africa (Raymo et al. 1996), and this wetter condition has driven the expansion of Lake Tanganyika (Meyer 1993), further opening new ecological niches that may have predisposed African Cyprinidae to the second speciation shift. The great depths of several African Lakes facilitated the persistence of fish lineages (most African Cyprinidae are benthic) during stressful environmental conditions (rifting, lake desiccation, mega-drought, etc.; Lippitsch 1997). In such conditions, species hybridized and the hybrid clades developed adaptive genetic (e.g., polyploidy) and ecological novelties. Indeed, Cyprinidae developed a wide range of polyploidy levels (Tsigenopoulos et al. 2002) that predispose for large ecological tolerance (Otto and Whitton 2000), a key evolutionary mechanism of speciation (Soltis and Soltis 1999). Additionally, several African *Labeobarbus* species have evolved unique anatomical features (Sibbing et al. 1998), piscivory (De Graaf et al. 2008) and lacustrine spawning (De Graaf et al. 2005) to survive harsh conditions. When the conditions became suitable (e.g., ~12–13 thousand years ago (ka)), lake levels filled to their current level (Stager and Johnson 2008), and the pre-adapted lineages may have shifted their speciation rate to fill new niches. However, around 2.0 Ma, Africa became drier (Trauth et al. 2005) and the extreme climatic variation in the last 500ka led to the desiccation of several African Lakes (Johnson et al. 1996), potentially leading to the sudden decrease of diversification of African Cyprinidae toward the present-day (Figure 3F).

Overall, the diversification rate of African Cyprinidae is much lower than that reported for African Cichlids. Most of diversification events of African Cyprinidae occurred in the last 10 million years following an episodic birth-death diversification model. This is in accord with what has been reported for Cyprinidae in the Asian freshwater (Yuan et al. 2010). Specifically, Yuan et al. (2010) reported that the speciation events of Cyprinidae in Chinese freshwaters likely occurred between 11.4 and 2.3 Mya, perhaps pointing to a simultaneous diversification at global scale.

In addition, 12 extinction events were observed during this diversification, supporting an earlier report that extinction event is frequent in freshwaters (Bloom et al. 2013). All these events could have been mediated through geological events and historical climate fluctuations on the continents (Danley et a. 2012; Chakona et al. 2013). As we should caution against interpreting evolution events based on a single analysis, we also tested our findings assessing their sensitivity to an automatic empirical hyperprior, and we found consistency for the findings reported in the present study. However, one potential caveat to this study is that it relies solely on a mitochondrial marker which could potentially show saturation and thus underestimate the branch lengths towards the origin of the phylogenetic tree (Revell et al. 2005). If this is true for the phylogeny of African Cyprinidae based on COI marker alone, we should expect a burst of speciation at the base of the radiation because of underestimation of older branch lengths. Our results did not corroborate a burst of speciation towards the origin of diversification, thus undermining potential bias due to the use of COI alone.

Nonetheless, although we do not foresee any reason why the marker used may blur the diversification pattern, it is important to remind us that the present study is based on a single gene marker and that “type 2” species outnumber the “type 1”. The study should therefore be regarded as a basis for further investigation. We call for more studies that should use more markers to revisit the diversification patterns reported in this study.

## Supplementary materials

Supplementary material 1 Phylogenetic tree used in the present analysis Data type: molecular data Explanation note: Paper currently in review in which we have presented the phylogenetic tree of African Cyprinidae. We submit it here as supplementary Information because we do not want to publish the same phylogeny twice. This is an open access article distributed under the terms of the Creative Commons Attribution License (CC BY 4.0), which permits unrestricted use, distribution, and reproduction in any medium, provided the original author and source are credited.Mariam I. Adeoba, Kowiyou Yessoufou
