# Supplementary figures and images for: Analysis of temporal diversification of African Cyprinidae (Teleostei, Cypriniformes)
Source: Zookeys. 2018 Dec 13;(806):141–61. doi: 10.3897/zookeys.806.25844 (PMC6302146; doi:10.3897/zookeys.806.25844)

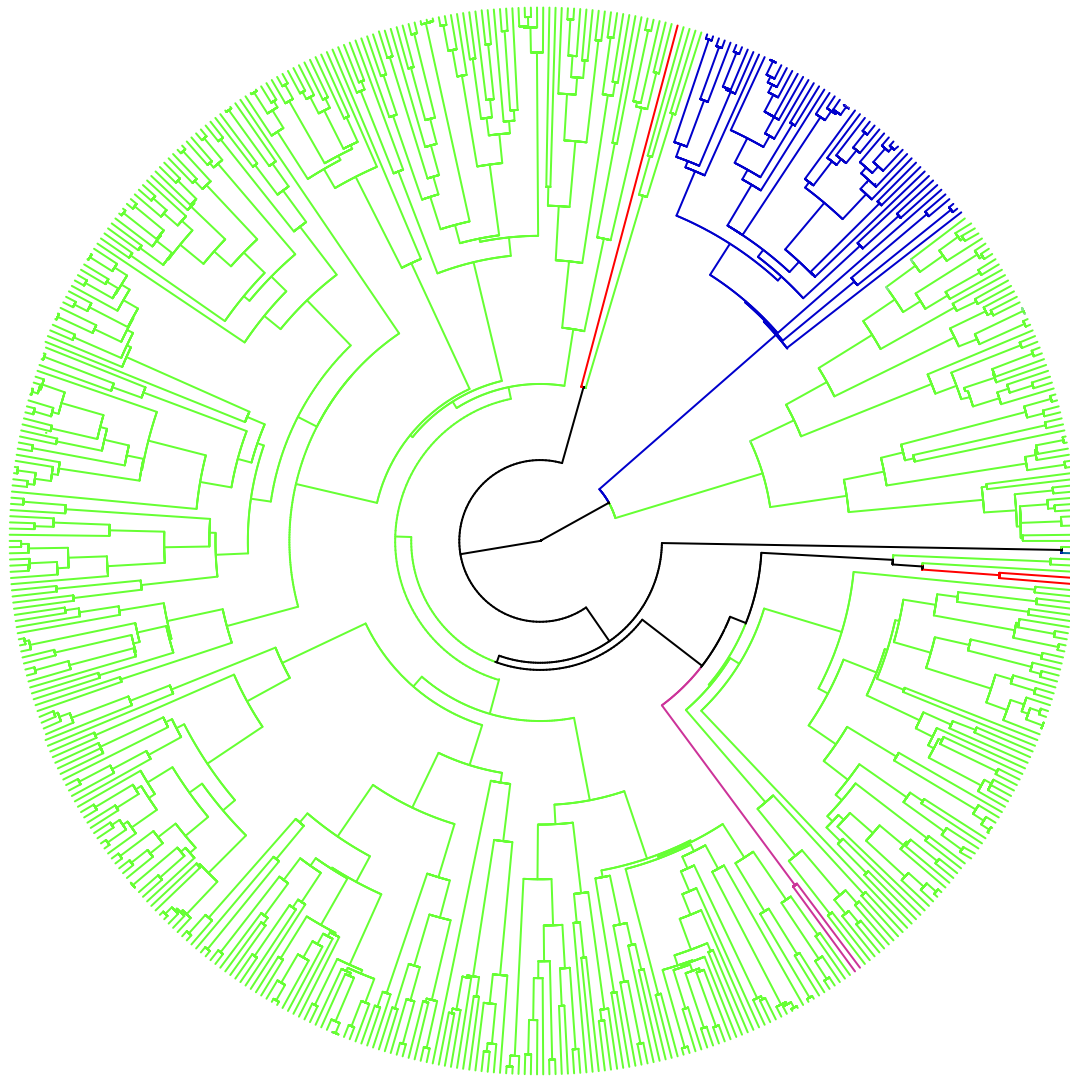

| Subfamilies |
|-------------|
| Cyprininae  |
| Danioninae  |
| Leucisinae  |
| Not-defined |

Supplement: Supplementary material 1 — Phylogenetic tree used in the present analysis [file zookeys-806-141-s001.pdf]
